# Aerobic Exercise-Mediated Regulation of Ferroptosis in Skeletal Disorders: Molecular Mechanisms and Potential Applications

**DOI:** 10.3390/biom16081210

**Published:** 2026-08-19

**Authors:** Rui Pu, Guo-Pan Gong, Wen-Li Song, Yue Yin, Zi-Yang Chen, Pan Jin

**Affiliations:** 1 Laboratory of Kinesiology and Human Sport Science, College of Education and Sports Sciences, Yangtze University, Jingzhou 434023, China; purui@yangtzeu.edu.cn (R.P.);; 2 Health Science Center, Yangtze University, Jingzhou 434023, China

**Keywords:** aerobic exercise, ferroptosis, skeletal disorders, osteoblasts, osteoclasts, bone homeostasis

## Abstract

Skeletal disorders, including osteoporosis, osteoarthritis, rheumatoid arthritis, and osteonecrosis of the femoral head, are common chronic conditions that substantially affect health and quality of life. Ferroptosis, a form of regulated cell death driven by iron-dependent lipid peroxidation, has increasingly been implicated in abnormal bone remodeling, cartilage degeneration, synovial pathology, and impaired skeletal homeostasis. Aerobic exercise is an important non-pharmacological approach for maintaining skeletal health, but the role of ferroptosis in its protective effects remains incompletely understood. Previous reviews have mainly discussed ferroptosis in skeletal disorders or the beneficial effects of exercise on skeletal health as separate topics. In contrast, this review places aerobic exercise, ferroptosis, and skeletal disorders within a unified framework and summarizes current evidence across osteoporosis, osteoarthritis, rheumatoid arthritis, and osteonecrosis of the femoral head. We further discuss how aerobic exercise may influence ferroptosis through the regulation of iron homeostasis, lipid peroxidation, antioxidant defense, and inflammatory responses, with attention to recently emerging molecular evidence and to the distinction between direct findings from bone- and joint-related tissues and supportive evidence from non-skeletal systems. Current direct evidence is concentrated mainly in osteoblast-related bone loss and osteoarthritis and is derived predominantly from animal and cellular studies, whereas direct clinical evidence in humans remains limited. Overall, available evidence supports ferroptosis as a potential mechanistic link between aerobic exercise and skeletal protection, but its role in mediating exercise-induced benefits in humans has yet to be established. Further clinical validation of this relationship may help clarify the biological basis of aerobic exercise interventions and support the development of more targeted exercise strategies for the prevention and management of skeletal disorders.

## 1. Introduction

Bone metabolism is a dynamic process maintained by the coordinated activities of bone-forming osteoblasts (OBs), bone-resorbing osteoclasts (OCs), and osteocytes, which regulate bone remodeling through paracrine signaling [1]. Under physiological conditions, the number and function of OCs and OBs remain tightly balanced, thereby maintaining bone metabolic homeostasis. Disruption of this balance may lead to skeletal disorders, including osteoporosis (OP), osteoarthritis (OA), rheumatoid arthritis (RA), and osteonecrosis of the femoral head (ONFH) [2,3,4]. With global population aging, the burden of age-related diseases continues to increase, and the burden of skeletal disorders is rising worldwide [5,6]. For example, a systematic review and meta-analysis involving 343,704 participants estimated the global prevalence of osteoporosis at approximately 19.7% [6], while approximately 595 million people worldwide were living with osteoarthritis in 2020, accounting for 7.6% of the global population [7]. These disorders are usually chronic and disabling, seriously reducing patients’ quality of life and imposing an increasing economic burden on healthcare systems. Although conventional pharmacological interventions are widely used, their long-term application may be associated with adverse effects. Therefore, identifying safer non-pharmacological interventions that target bone cell function has become an important focus in bone biology [8].

The regulation of cell death has long been a central topic in life science research. In recent years, ferroptosis, a newly recognized form of regulated cell death characterized by iron-dependent lipid peroxide accumulation, has attracted increasing attention [9,10]. Ferroptosis is mainly associated with disordered iron metabolism, impaired antioxidant defense, and excessive lipid peroxide accumulation [11]. Increasing evidence indicates that ferroptosis-related regulatory pathways exist in multiple bone-related cells, including OBs, OCs, and chondrocytes. Experimental evidence indicates that aberrant activation of ferroptosis can impair cellular function and may contribute to the pathological progression of skeletal disorders [12,13,14,15]. For example, ferroptosis in OBs and OCs is closely associated with OP; iron deposition-induced chondrocyte ferroptosis may contribute to OA; inflammation-related ferroptosis may accelerate joint destruction in RA; and inhibition of ferroptosis may promote osteogenic differentiation and delay the progression of ONFH. These findings suggest that targeting ferroptosis in bone tissue may represent a potential strategy for the prevention and treatment of skeletal disorders.

Aerobic exercise has multiple biological effects, including promoting bone formation, maintaining bone metabolic homeostasis, enhancing antioxidant capacity, and reducing lipid peroxidation, and has gradually become a research focus in the field of bone health [16,17,18]. The present review focuses specifically on aerobic exercise because current evidence linking exercise to ferroptosis regulation is predominantly derived from aerobic exercise paradigms. Although resistance, high-impact, and combined exercise are also widely used to improve skeletal health, studies directly examining their effects on ferroptosis remain comparatively limited. Therefore, whether different exercise modalities exert distinct regulatory effects on ferroptosis has yet to be established. In addition, aerobic exercise encompasses modalities such as walking that are relatively accessible, require limited specialized equipment, and allow exercise intensity and duration to be readily individualized according to functional status and exercise tolerance [19,20]. These characteristics may enhance the feasibility and translational potential of aerobic exercise as a non-pharmacological approach for maintaining skeletal health. Recent experimental studies suggest that the protective effects of aerobic exercise on skeletal tissues may be partly related to the regulation of ferroptosis. However, current findings remain relatively scattered, and the regulatory pathways involved in different skeletal cell types have not been systematically integrated. Moreover, direct evidence linking aerobic exercise to ferroptosis regulation in skeletal tissues remains limited, while part of the available mechanistic evidence is derived from non-skeletal experimental systems. Therefore, based on the mechanisms of ferroptosis, this review further explores the molecular relationships among aerobic exercise, ferroptosis, and skeletal disorders from four aspects: osteogenic differentiation, cartilage degradation, inflammatory regulation, and antioxidant defense. A distinctive aspect of this review is the integration of current evidence on the aerobic exercise–ferroptosis–skeletal disorder axis, with particular attention to distinguishing direct skeletal evidence from mechanistic evidence derived from non-skeletal tissues. This review aims to clarify the current evidence linking aerobic exercise, ferroptosis, and skeletal disorders and to identify disease-specific questions that require further investigation.

## 2. Literature Search Strategy

A narrative literature search was conducted using PubMed, the Web of Science Core Collection, and Embase to identify studies relevant to ferroptosis, aerobic exercise, and skeletal disorders. The literature search was continuously updated during manuscript preparation and revision and was last updated in August 2026. No lower publication-date limit was imposed.

Search terms were used individually and in relevant combinations and included terms related to ferroptosis (“ferroptosis”, “iron metabolism”, “lipid peroxidation”, “GPX4”, “SLC7A11”, “Nrf2”, and “ACSL4”), aerobic exercise (“exercise”, “aerobic exercise”, “treadmill”, “endurance exercise”, “training”, “running”, and “swimming”), and skeletal disorders or skeletal cell types (“osteoporosis”, “osteoarthritis”, “rheumatoid arthritis”, “osteonecrosis of the femoral head”, “osteoblast”, “osteoclast”, “osteocyte”, “chondrocyte”, and “synovial cell”).

Primary experimental studies directly addressing ferroptosis in skeletal cells, tissues, or disease models and studies investigating exercise-mediated regulation of ferroptosis were prioritized. Review articles and other authoritative publications were used primarily to provide background information and to identify relevant primary studies. The included evidence was interpreted according to the experimental level, including in vitro studies, preclinical animal studies, and human investigations. Particular attention was also given to the tissue origin of the evidence. Studies conducted in osteoblasts, osteoclasts, osteocytes, chondrocytes, synovial cells, bone or joint tissues, or established models of skeletal disorders were considered direct skeletal evidence. Where direct skeletal evidence was limited, mechanistically relevant findings from skeletal muscle, cardiovascular, neural, hepatic, or other non-skeletal experimental systems were considered as supportive mechanistic evidence. Such findings were explicitly distinguished from direct skeletal evidence and were not interpreted as direct proof that the same ferroptosis-regulatory mechanisms occur in skeletal tissues. Studies that did not provide relevant information on ferroptosis, aerobic exercise, or the skeletal disorders covered in this review were not used as primary mechanistic evidence.

## 3. Mechanisms of Ferroptosis

Ferroptosis is an iron-dependent form of regulated cell death characterized by excessive phospholipid peroxidation and failure of cellular antioxidant defense. Unlike apoptosis and other forms of regulated cell death, ferroptosis is closely associated with disturbances in iron metabolism, membrane lipid peroxidation, and antioxidant defense systems [21,22,23]. Perturbation of intracellular iron homeostasis can markedly increase ferroptosis susceptibility. Transferrin receptor 1 (TFRC) mediates cellular iron uptake and contributes to the intracellular iron pool that influences ferroptosis susceptibility [24]. Intracellular iron is normally stored in ferritin, whereas nuclear receptor coactivator 4 (NCOA4)-mediated ferritinophagy promotes ferritin degradation and releases stored iron, thereby increasing the labile Fe^2+^ pool and enhancing ferroptosis susceptibility [25,26]. Excess Fe^2+^ further promotes the generation of highly reactive hydroxyl radicals through the Fenton reaction, thereby enhancing oxidative damage and lipid peroxidation [21,27].

The execution of ferroptosis ultimately depends on the peroxidation of membrane phospholipids. Polyunsaturated fatty acid-containing phospholipids (PUFA-PLs) are particularly susceptible to oxidation [21,27]. Acyl-CoA synthetase long-chain family member 4 (ACSL4) activates long-chain PUFAs, whereas lysophosphatidylcholine acyltransferase 3 (LPCAT3) facilitates their incorporation into membrane phospholipids. These processes increase the abundance of oxidizable PUFA-PLs and promote phospholipid hydroperoxide accumulation [28,29]. Additional oxidoreductases, including cytochrome P450 oxidoreductase (POR) and cytochrome b5 reductase 1 (CYB5R1), may further facilitate phospholipid peroxidation [30,31]. Lipid peroxidation generates secondary products such as 4-hydroxynonenal (4-HNE) and malondialdehyde (MDA), which reflect oxidative lipid damage [27]. Pharmacological inhibition of ferroptosis with ferrostatin-1 has also been shown to reduce lipid peroxidation and oxidative tissue injury in experimental models, further supporting lipid peroxidation as a major component of ferroptotic damage [32].

The System Xc^−^–GSH–GPX4 axis is the best-characterized antioxidant defense pathway against ferroptosis. System Xc^−^, composed of solute carrier family 7 member 11 (SLC7A11) and solute carrier family 3 member 2 (SLC3A2), mediates cystine uptake and provides cysteine for glutathione (GSH) synthesis. GPX4 subsequently uses GSH to reduce phospholipid hydroperoxides to non-toxic lipid alcohols, thereby preventing the propagation of lipid peroxidation [22,23,33]. Beyond its role in ferroptosis suppression, GPX4-mediated control of lipid peroxidation can also influence inflammatory signaling. For example, GPX4 deficiency enhances lipid peroxidation and promotes stimulator of interferon gene (STING) carbonylation, thereby impairing STING activation [34]. In addition to GPX4, several parallel antioxidant systems have been identified. FSP1 suppresses ferroptosis through the NAD(P)H–coenzyme Q10 (CoQ10) pathway [35], whereas the GTP cyclohydrolase 1 (GCH1)–tetrahydrobiopterin (BH4) system provides another GPX4-independent mechanism that protects membrane phospholipids against oxidation [36]. DHODH may additionally contribute to mitochondrial ferroptosis defense in specific cellular contexts [37].

**Figure 1:**
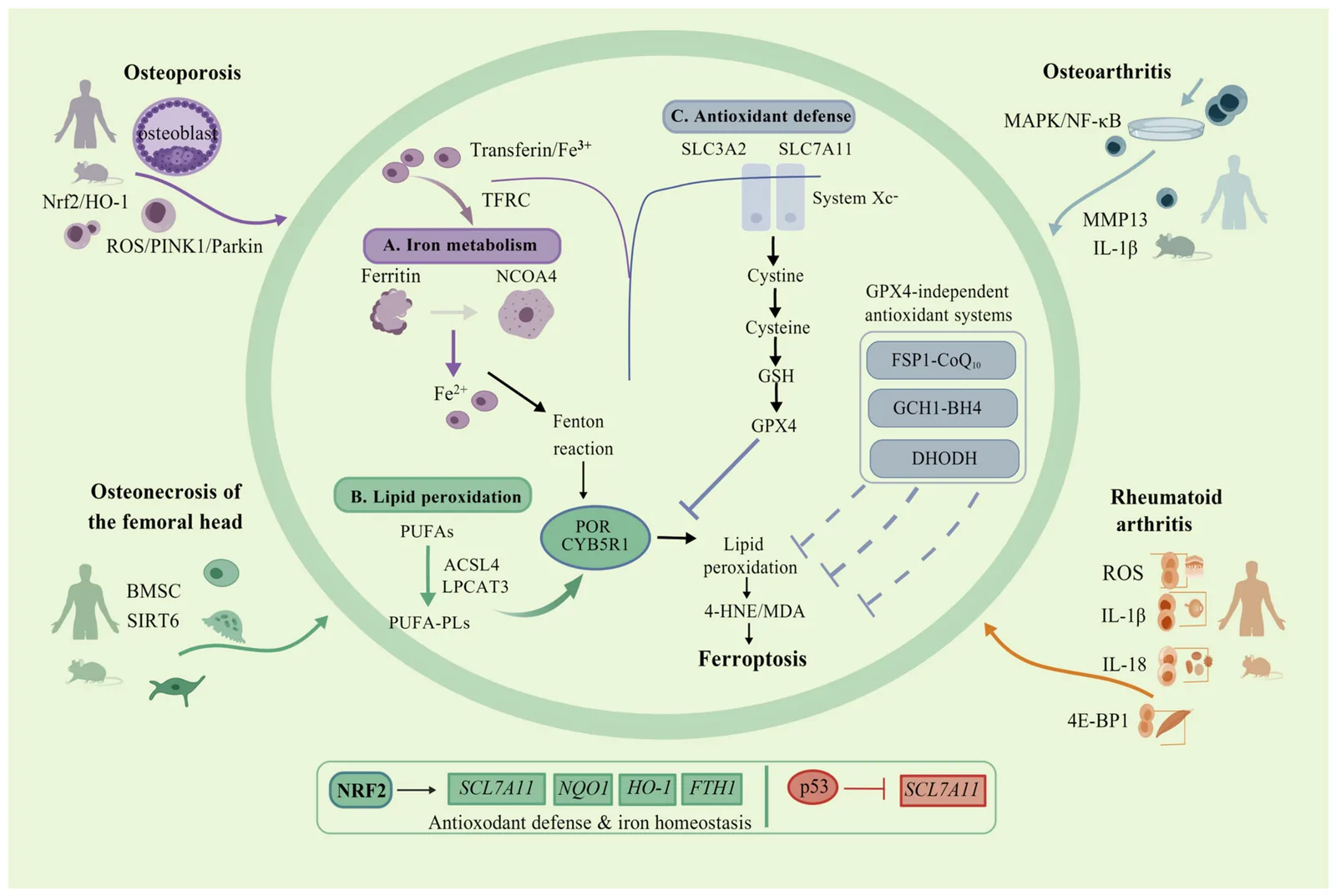
Major mechanisms of ferroptosis and ferroptosis-related changes in skeletal disorders. Ferroptosis is driven by iron-dependent lipid peroxidation and is counteracted by cellular antioxidant defenses. TFRC-mediated iron uptake and NCOA4-mediated ferritinophagy contribute to the intracellular Fe^2+^ pool, which promotes lipid oxidation through the Fenton reaction. ACSL4/LPCAT3-mediated phospholipid remodeling and the oxidoreductases POR and CYB5R1 further facilitate the peroxidation of PUFA-containing phospholipids. The System Xc^−^–GSH–glutathione peroxidase 4 (GPX4) axis provides the major antioxidant defense against ferroptosis, together with GPX4-independent systems involving FSP1–CoQ10, GCH1–BH4, and dihydroorotate dehydrogenase (DHODH). Nuclear factor erythroid 2-related factor 2 (Nrf2) regulates several genes involved in antioxidant defense and iron homeostasis, including SLC7A11, NQO1, HO-1, and FTH1, whereas p53 can increase ferroptosis susceptibility by repressing SLC7A11. Representative ferroptosis-related alterations reported in osteoporosis, osteoarthritis, rheumatoid arthritis, and osteonecrosis of the femoral head are shown around the central regulatory network.

Several stress-responsive transcriptional regulators also modify cellular susceptibility to ferroptosis. Nrf2 modulates cellular antioxidant and iron-handling capacity by regulating genes involved in redox homeostasis and iron metabolism, including SLC7A11, NAD(P)H quinone oxidoreductase 1 (NQO1), heme oxygenase-1 (HO-1), and ferritin heavy chain 1 (FTH1) [38]. Alterations in hypoxia-inducible factor-1 alpha (HIF-1α)/HO-1 signaling have also been associated with iron accumulation, oxidative stress, and ferroptosis in experimental disease models [39]. In contrast, p53 can promote ferroptosis under certain conditions by repressing SLC7A11 and limiting cystine uptake, although its effects are dependent on cellular context [40]. Taken together, ferroptosis susceptibility reflects the balance between iron availability and phospholipid peroxidation on the one hand and cellular antioxidant defense on the other (Figure 1). These mechanisms provide the molecular basis for understanding the involvement of ferroptosis in skeletal disorders and its potential regulation by aerobic exercise.

## 4. The Role of Ferroptosis in the Development of Skeletal Disorders

Recent studies have shown that ferroptosis is an important pathological mechanism involved in skeletal disorders and plays a key role in the regulation of bone metabolism. The following section summarizes the effects of ferroptosis on different skeletal disorders (Table 1).

### 4.1. Ferroptosis and OP

OP is a systemic skeletal disease characterized by decreased bone mineral density and bone mass, deterioration of bone microarchitecture, increased bone fragility, and a higher risk of fracture [41]. Osteoblast ferroptosis is an important contributor to bone loss in OP. In patients with OP, the deposition of advanced glycation end products in bone tissue increases the levels of MDA and free iron in OBs, while reducing glutathione (GSH) levels. These changes may induce OB ferroptosis and contribute to the development of OP [42]. Melatonin has been shown to activate the Nrf2/HO-1 signaling pathway, thereby inhibiting high-glucose-induced OB ferroptosis and enhancing the osteogenic differentiation capacity of MC3T3-E1 cells. This effect may improve bone microstructure and reduce the risk of OP [43]. These findings indicate that OB ferroptosis is closely associated with OP, suggesting that inhibition of ferroptosis may represent a potential therapeutic strategy for OP.

Studies on diabetic OP have shown that, under high-glucose conditions, overexpression of mitochondrial ferritin (FtMt) reduces oxidative stress caused by excessive ferrous ions and inhibits OB ferroptosis. Further evidence indicates that FtMt deficiency enhances reactive oxygen species (ROS) accumulation and activates PTEN-induced kinase 1 (PINK1)/Parkin-mediated mitophagy, thereby aggravating ferroptosis in osteoblasts [44]. These findings suggest that upregulation of FtMt may reduce iron overload and Fenton reaction-derived ROS, thereby inhibiting ferroptosis and reducing cellular injury. In contrast, FtMt deficiency may negatively affect OB survival through mitophagy-related mechanisms. Therefore, modulation of FtMt-mediated ferroptosis may be a potential therapeutic target for type 2 diabetes-associated OP.

In addition to OBs, ferroptosis in OCs is also involved in OP progression. 2-Methoxyestradiol (2-ME2) induces ferroptosis in OCs by regulating HIF-1α and ferritin, thereby inhibiting OC-mediated bone resorption and slowing the pathological progression of OP [45]. In addition, the osteoclast differentiation regulator aconine significantly reduces the expression of cathepsin K (CTSK) and matrix metalloproteinase-9 (MMP9) in OCs, inhibits osteoclastogenesis, and reduces excessive bone resorption. Aconine also promotes GPX4 expression by regulating the phosphorylation of inhibitor of κB (IκB) and p65, while suppressing the production of acyl-CoA synthetase long-chain family member 4 (ACSL4) in OCs [46]. However, current evidence linking ferroptosis to OP is derived predominantly from animal and cellular models, and its contribution to human OP remains to be further established.

### 4.2. Ferroptosis and OA

OA is mainly characterized by degenerative changes in articular cartilage, accompanied by synovial hyperplasia, osteophyte formation, and narrowing of the joint space [47]. Human tissue and experimental evidence indicate that GPX4 expression is reduced in OA cartilage, and decreased GPX4 increases the susceptibility of chondrocytes to oxidative stress and ferroptosis. This may aggravate extracellular matrix (ECM) degradation through mitogen-activated protein kinase (MAPK)/nuclear factor-kappa B (NF-κB)-related signaling and contribute to OA progression [48]. Ferroptosis is also involved in abnormal subchondral bone remodeling. In obesity-related OA models, activation of the p53–protein kinase B (AKT)–forkhead box O3 (FOXO3) axis is associated with ferroptosis-related osteoclast differentiation and altered expression of ferroptosis suppressors, including GPX4, SLC7A11, and ferroptosis suppressor protein 1 (FSP1). Modulation of this pathway attenuates abnormal subchondral bone remodeling and OA progression [49]. In addition, systemic iron overload can increase joint iron accumulation and inflammatory mediators, including interleukin-1 beta (IL-1β) and tumor necrosis factor (TNF), thereby aggravating cartilage degeneration [50]. Although this study did not directly establish ferroptosis, it supports iron dysregulation as a pathological condition that may increase ferroptosis susceptibility in OA.

Conversely, inhibition of ferroptosis may exert protective effects against cartilage degeneration. Ferrostatin-1 (Fer-1), a ferroptosis inhibitor, has been shown to alleviate chondrocyte injury and OA progression by suppressing ferroptosis [51]. Animal studies have further shown that Fer-1 increases type II collagen levels, reduces matrix metalloproteinase-13 (MMP13) expression, suppresses inflammatory responses, and alleviates cartilage degeneration in mouse models of OA [52]. In addition, reduced 4-HNE levels in the knee cartilage and meniscus of iron-deficient animals were closely associated with iron concentration. Lowering iron levels may reduce ROS production, prevent lipid peroxidation, and decrease the risk of OA progression [53]. Collectively, current evidence indicates that ferroptosis may contribute to OA through multiple cellular compartments, including chondrocyte injury and osteoclast-associated subchondral bone remodeling. Although human OA tissues show ferroptosis-related molecular alterations, most causal evidence remains derived from animal and cellular models.

### 4.3. Ferroptosis and RA

RA is a chronic inflammatory joint disease characterized by synovial hyperplasia, pannus formation, progressive cartilage destruction, bone erosion, and loss of joint function [54]. Compared with healthy individuals, patients with RA exhibit increased oxidative stress and elevated ROS levels in the joint microenvironment [55]. ROS can promote lipid peroxidation and activate inflammatory pathways, including the NLR family pyrin domain containing 3 (NLRP3) inflammasome, leading to increased production of IL-1β and interleukin-18 (IL-18) [56,57]. Disturbances in iron metabolism and macrophage iron handling may further amplify oxidative stress within the inflammatory microenvironment [58,59,60]. These conditions provide a biological environment that may favor ferroptosis, although evidence from these studies alone does not establish ferroptosis as a direct driver of RA.

More direct evidence has been obtained in synovial fibroblasts, a major pathological cell population in RA. Analysis of RA synovial tissues and experimental arthritis models has shown increased lipid peroxidation and iron accumulation, whereas TNF signaling enhances the resistance of synovial fibroblasts to ferroptosis by promoting SLC7A11-dependent cystine uptake and glutathione synthesis. Pharmacological induction of ferroptosis reduced pathological synovial fibroblast accumulation and attenuated arthritis severity in experimental models [61]. Similarly, semaphorin 5A activates the phosphoinositide 3-kinase (PI3K)/AKT/mechanistic target of rapamycin (mTOR) pathway and increases eukaryotic translation initiation factor 4E-binding protein 1 (4E-BP1) phosphorylation, thereby suppressing lipid peroxidation and ferroptosis in synovial fibroblasts [62]. In contrast, the bioactive peptide galectin-1-derived peptide 3 promotes ferroptosis in MH7A cells through the p53/SLC7A11 axis, further supporting the potential of ferroptosis induction to restrict pathological synovial fibroblast survival [63].

These findings indicate that the role of ferroptosis in RA is cell- and context-dependent: ferroptosis may contribute to oxidative tissue injury in the inflammatory joint microenvironment, whereas ferroptosis resistance in pathological synovial fibroblasts may facilitate their persistence. Thus, therapeutic modulation of ferroptosis in RA may require cell-specific rather than uniformly inhibitory strategies. However, current mechanistic evidence remains predominantly preclinical, and its therapeutic relevance in patients with RA requires further validation.

### 4.4. Ferroptosis and ONFH

ONFH is a complex disease involving multiple pathological processes, including abnormal bone metabolism, lipid metabolism imbalance, vascular dysfunction, and impaired microcirculation in the femoral head [64]. Experimental evidence indicates that ferroptosis contributes to steroid-induced ONFH. In a steroid-induced ONFH rat model and in bone marrow mesenchymal stem cell (BMSC) experiments, exogenous melatonin suppressed ROS accumulation and lipid peroxidation and restored ferroptosis-related antioxidant proteins, including GPX4 and SLC7A11, through growth differentiation factor 15 (GDF15)-mediated signaling. These effects preserved BMSC viability and osteogenic capacity and attenuated femoral head injury [65]. These findings support a protective role of melatonin-mediated ferroptosis inhibition in steroid-induced ONFH.

Sirtuin 6 (SIRT6) is involved in the regulation of bone and vascular homeostasis [66]. In glucocorticoid-induced ONFH models, SIRT6 overexpression increases GPX4 and SLC7A11 expression, restores GSH levels, and reduces ROS and MDA accumulation, thereby suppressing ferroptosis in vascular endothelial cells. These changes are accompanied by improved angiogenic and osteogenic responses in the femoral head [67]. This finding indicates that ferroptosis in ONFH may involve not only impaired osteogenesis but also vascular injury, which is consistent with the important role of microcirculatory dysfunction in disease progression.

In addition, integrated bioinformatics, human tissue proteomics, and cellular validation identified ferroptosis-related biomarkers, including neutrophil cytosolic factor 2 (NCF2) and solute carrier family 2 member 1 (SLC2A1), in steroid-induced ONFH. Subsequent cellular experiments further suggested that inhibition of ferroptosis can preserve osteogenic differentiation under glucocorticoid-induced stress [68]. Overall, current evidence supports an association between ferroptosis, impaired osteogenesis, and vascular dysfunction in steroid-induced ONFH. However, compared with OP and OA, mechanistic evidence remains limited and is based largely on animal, cellular, and omics studies rather than interventional clinical investigations. Further studies are therefore needed to determine the contribution of ferroptosis to human ONFH and its potential therapeutic relevance.

**Table 1:** Experimental evidence for the involvement of ferroptosis in skeletal disorders.

Diseases	Experimental Model	Target Tissue/Cell	Ferroptosis-Related Markers/Pathways	Main Findings	Evidence Level	Ref.
OP	High-glucose-treated MC3T3-E1 cells with ferroptosis-related intervention	Osteoblasts	Nrf2/HO-1; ferroptosis-related oxidative stress	Inhibition of high glucose-induced ferroptosis in OBs enhances osteogenic capacity.	In vitro	[43]
OP	High-fat-diet-induced diabetic/osteoporotic rat model with FtMt-related intervention	Bone tissue; osteoblasts	FtMt; ROS; PINK1/Parkin; mitophagy	FtMt deficiency promotes mitophagy-associated osteoblast ferroptosis and aggravates bone loss.	Animal + in vitro	[44]
OP	Osteoporosis-related mouse model with aconine intervention	Osteoclasts; bone tissue	NF-κB; GPX4; ACSL4	Ferroptosis inhibition suppresses osteoclast-mediated bone resorption.	Animal + cellular	[46]
OA	Experimental OA mice treated with Fer-1/DFO	Articular cartilage; chondrocytes	GPX4; lipid peroxidation; MAPK/NF-κB	Reduced GPX4 increases chondrocyte susceptibility to ferroptosis and ECM degradation.	Animal + cellular	[48]
OA	HFD-induced obesity-related OA mice, human OA joint samples, and cellular models	Subchondral bone; osteoclasts; BMSCs	p53–AKT–FOXO3; GPX4; SLC7A11; FSP1; Fe^2+^; ROS/lipid peroxidation	Obesity-related OA promotes ferroptosis-associated osteoclast differentiation and abnormal subchondral bone remodeling. Targeting the p53–FOXO3 axis modulates osteoclast ferroptosis and attenuates OA progression.	Human tissue + animal + in vitro	[49]
OA	Experimental OA mice and chondrocytes	Articular cartilage; chondrocytes	GPX4; Nrf2; MMP13; collagen II	Chondrocyte ferroptosis contributes to cartilage degeneration and OA progression.	Animal + cellular	[52]
RA	RA patient synovial samples, CIA mice, and synovial fibroblast models	Synovium; synovial fibroblasts	TNF; SLC7A11; GCLC/GCLM; GSH; GPX4; lipid peroxidation	TNF signaling promotes ferroptosis resistance in synovial fibroblasts by enhancing cystine uptake and GSH synthesis. Pharmacological induction of ferroptosis reduces pathological fibroblast accumulation and attenuates arthritis progression.	Human tissue + animal + in vitro	[61]
RA	Synovial fluid and synovial tissue from patients with RA/OA and cultured synovial fibroblasts	Synovium; synovial fibroblasts	SEMA5A; PI3K/AKT/mTOR; 4E-BP1; GPX4; SREBP1/SCD1	SEMA5A activates PI3K/AKT/mTOR signaling and suppresses ferroptosis in synovial fibroblasts, contributing to the survival of pathological fibroblasts in RA.	Human tissue + in vitro	[62]
RA	TNF-α-treated MH7A cells	Fibroblast-like synoviocyte cell line	p53/SLC7A11; GSH/GPX4	Galectin-1-derived peptide 3 induces ferroptosis through p53-mediated suppression of SLC7A11, providing a potential strategy for targeting RA-FLS-like cells.	In vitro	[63]
ONFH	Steroid-induced ONFH rat model and BMSC experiments	Femoral head; BMSCs	MT2/GDF15; GPX4; SLC7A11; ROS/lipid peroxidation	Melatonin suppresses ferroptosis through GDF15-related signaling, preserves BMSC viability and osteogenic capacity, and attenuates steroid-induced ONFH.	Animal + in vitro	[65]
ONFH	Glucocorticoid-induced ONFH rat model and endothelial cell experiments	Femoral head; vascular endothelial cells	SIRT6; GPX4; SLC7A11; GSH; ROS/MDA	SIRT6 suppresses ferroptosis, improves angiogenic and osteogenic responses, and alleviates glucocorticoid-induced femoral head injury.	Animal + in vitro	[67]
ONFH	Steroid-induced ONFH transcriptomic datasets, human necrotic/healthy tissue validation, and cell experiments	Femoral head tissue; osteogenic cells	NCF2; SLC2A1; ferroptosis-related genes	Ferroptosis-related biomarkers were identified and validated, and inhibition of ferroptosis was associated with improved osteogenic differentiation.	Human tissue/proteomics + bioinformatics + in vitro	[68]

## 5. Ferroptosis in the Effects of Aerobic Exercise on Skeletal Disorders

### 5.1. Effects of Aerobic Exercise on Ferroptosis

Iron is essential for collagen synthesis, vitamin D activation, and normal skeletal homeostasis [69]. Experimental studies indicate that exercise can influence iron-related physiological processes. Twelve weeks of running exercise increased the bone formation marker procollagen type I *N*-terminal propeptide (PINP) in iron-adequate female rats [70], while strenuous exercise increased transferrin receptor expression and transferrin-mediated iron uptake in bone marrow erythroblasts [71]. Although these observations do not constitute direct evidence of exercise-mediated ferroptosis regulation, they indicate that exercise can modify iron handling and provide a biological basis for considering iron homeostasis as one potential link between exercise and ferroptosis.

Experimental evidence for exercise-mediated ferroptosis regulation has also been obtained from cardiovascular and neurological models. In doxorubicin-induced cardiotoxicity, endurance exercise preconditioning reduced Fe^2+^ accumulation and oxidative stress, improved mitochondrial function, and attenuated myocardial ferroptosis, with AMP-activated protein kinase alpha 2 (AMPKα2) activation and phosphorylation contributing to these effects. Swimming training was also shown to increase *miR-17-3p* expression, activate Kelch-like ECH-associated protein 1 (Keap1)/Nrf2 signaling, and suppress cyclic GMP–AMP synthase (cGAS)/STING signaling, accompanied by increased GPX4, SLC7A11, and GSH levels and reduced Fe^2+^ and MDA accumulation [72,73]. In APP/PS1 mice, 8 weeks of aerobic exercise activated the System Xc^−^/GPX4 pathway in the prefrontal cortex, reduced lipid peroxidation, and attenuated ferroptosis associated with iron overload [74]. Aerobic exercise further increased fibroblast growth factor 21 (FGF21), fibroblast growth factor receptor 1 (FGFR1), and peroxisome proliferator-activated receptor gamma coactivator 1-alpha (PGC-1α) expression and maintained AMPKα phosphorylation in the hypothalamic paraventricular nucleus, together with reduced ROS and lipid peroxidation, improved iron homeostasis, and decreased neuronal ferroptosis [75]. Nrf2 and brain-derived neurotrophic factor (BDNF)/tropomyosin receptor kinase B (TrkB)-related signaling have also been proposed as a potential link between physical exercise and ferroptosis regulation in neurological disorders such as Parkinson’s disease [76], although this relationship still requires direct experimental validation.

Direct evidence in bone- and joint-related tissues remains comparatively limited. In monosodium iodoacetate (MIA)-induced osteoarthritis, moderate-intensity treadmill exercise reduced chondrocyte ferroptosis and cartilage matrix degradation. Complementary cellular experiments showed that moderate mechanical stress activated Nrf2, inhibited NF-κB p65 signaling, increased collagen type II alpha 1 chain (Col2A1), GPX4, and SLC7A11 expression, and reduced MMP13 and p53 expression [77]. These findings provide relatively direct evidence that exercise-related mechanical stimulation can regulate ferroptosis in joint tissue. Exercise-mediated ferroptosis regulation has also been demonstrated in skeletal muscle, where exercise training suppressed STING expression and reduced lipid peroxidation-associated ferroptosis and muscle atrophy in high-fat-diet (HFD)-fed mice [78]. Pharmacological manipulation of STING further supported its involvement in this response. However, findings from skeletal muscle should be considered supportive mechanistic evidence rather than direct evidence that the same pathway operates in bone or joint cells.

**Figure 2:**
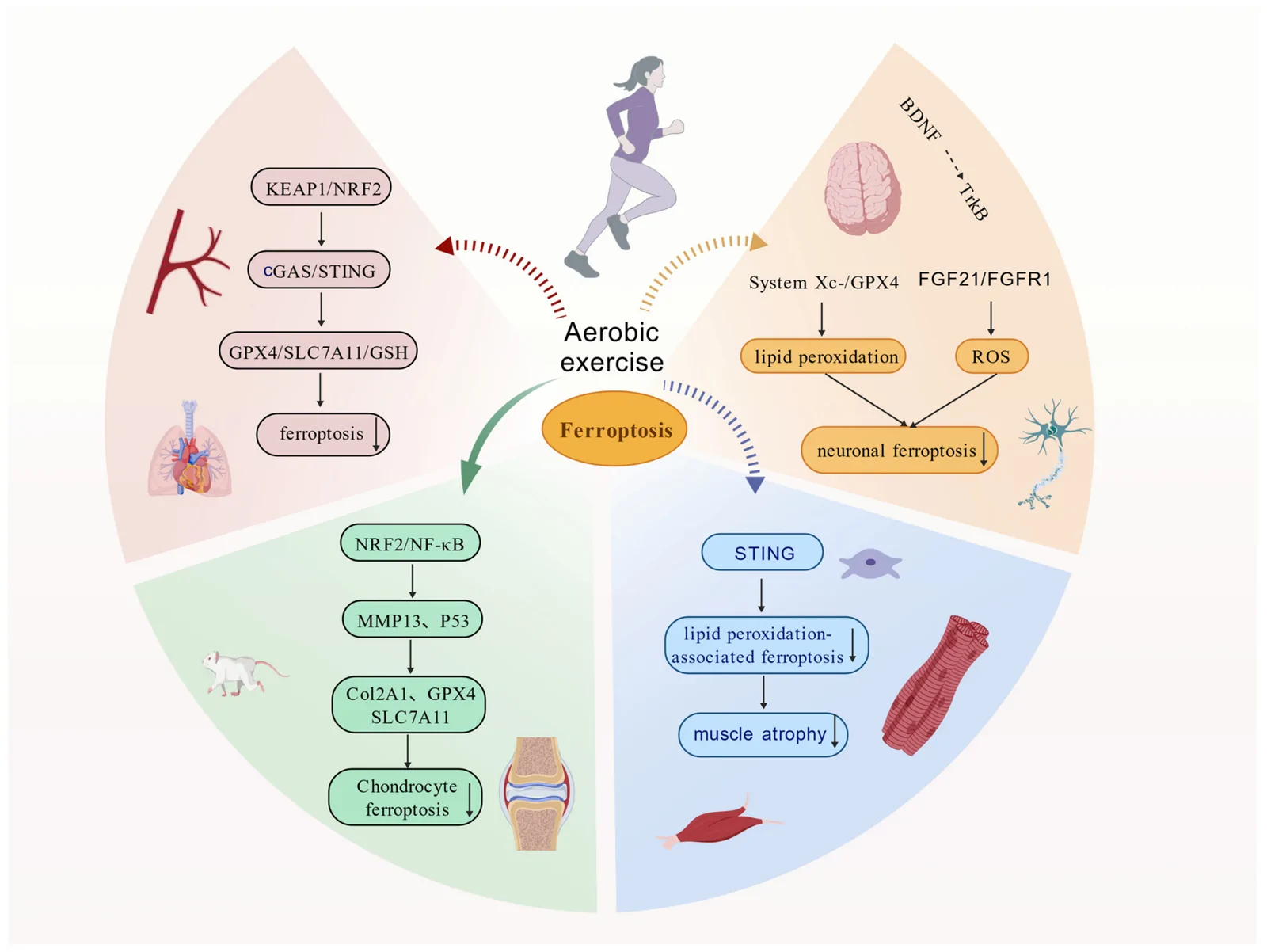
Effects of aerobic exercise on ferroptosis. Aerobic exercise may influence ferroptosis through the regulation of antioxidant defense, lipid peroxidation, iron homeostasis, and ferroptosis-related signaling pathways. Current direct skeletal evidence is mainly derived from cartilage and chondrocytes, whereas findings from cardiovascular, neurological, and skeletal muscle models provide supportive mechanistic evidence that requires tissue-specific validation before extrapolation to bone and joint tissues. Solid curved arrows indicate direct exercise-associated effects experimentally observed in skeletal tissues, whereas dashed curved arrows indicate indirect or downstream mechanistic effects derived mainly from non-skeletal tissues.

Overall, current evidence indicates that aerobic exercise can influence ferroptosis through interconnected changes in iron homeostasis, mitochondrial function, antioxidant defense, and lipid peroxidation (Figure 2). However, the evidence remains strongly tissue-dependent. Direct skeletal evidence is currently concentrated mainly in cartilage and chondrocytes, whereas several mechanisms involving AMPKα2, Keap1/Nrf2, System Xc^−^/GPX4, FGF21/FGFR1, BDNF/TrkB, and STING have been identified primarily in cardiovascular, neurological, or skeletal muscle models. These findings provide useful mechanistic clues but require tissue-specific validation before being extrapolated to bone and joint disorders. In addition, ferroptosis responses to exercise may vary with exercise modality, intensity, and duration, while direct comparisons among different exercise protocols remain scarce.

### 5.2. Potential Role of Ferroptosis in Aerobic Exercise-Mediated Improvement of Skeletal Disorders

Aerobic exercise has well-recognized beneficial effects on skeletal health and can influence bone turnover, bone formation, and bone homeostasis [79,80]. Mechanical stimulation associated with exercise can influence osteoclast differentiation and osteogenic signaling, including changes in bone resorption- and formation-related markers [81,82]. In addition, exercise training has been shown to improve bone metabolism-related indicators, including osteocalcin, in elderly women with osteoporosis [83]. Long-term aerobic exercise further increases bone formation-related biomarkers, including alkaline phosphatase (ALP) and osteocalcin (OCN), thereby contributing to improved bone health [18]. These findings suggest that aerobic exercise promotes osteoblast function, enhances mineral utilization such as calcium and phosphorus, and may delay the progression of OP. Osteoprotegerin (OPG) is an important regulator of bone homeostasis [84]. In ovariectomized mice, wheel-running exercise has been shown to suppress osteoclastogenesis and attenuate bone loss through CD8^+^ T-cell-derived interferon-gamma (IFN-γ) and inhibition of NF-κB and MAPK signaling [85]. Collectively, these studies establish beneficial effects of aerobic exercise on osteoblast–osteoclast balance and bone remodeling; however, they do not by themselves demonstrate that ferroptosis directly mediates these skeletal adaptations.

In OA, the chondroprotective effects of aerobic exercise may also be associated with ferroptosis regulation. In experimental joint and cartilage models, moderate-intensity aerobic exercise has been shown to reduce the expression of inflammatory and catabolic mediators such as IL-1β and MMP13, promote proteoglycan and collagen deposition, and restore cartilage metabolic balance, thereby exerting protective effects against OA progression [86]. Although these findings support the chondroprotective effects of exercise, ferroptosis was not necessarily evaluated as the direct mediator in all of these studies. Notably, studies in athletes engaged in high-intensity training have shown that excessive exercise may induce iron deficiency, leading to reduced hemoglobin and cytochrome oxidase levels, impaired cellular respiration, and metabolic dysfunction, ultimately resulting in decreased exercise performance [87]. Conversely, excessive iron accumulation observed in marathon runners may increase free radical generation and thereby promote oxidative stress and mitochondrial dysfunction, conditions that may increase susceptibility to ferroptosis [88]. These human observations reflect systemic alterations in iron metabolism rather than direct evidence of ferroptosis in bone or joint tissues. Nevertheless, they indicate that iron metabolic balance is important for both physiological adaptation and pathological responses associated with exercise.

Recent studies have shown that gallic acid (GA), a plant-derived phenolic compound, exhibits strong anti-inflammatory and antioxidant properties. In skeletal muscle, GA has been shown to improve mitochondrial function and redox homeostasis, including enhancement of intracellular ATP production and attenuation of exercise-induced mitochondrial oxidative stress [89,90]. Moreover, through its antioxidant capacity and ability to reduce iron accumulation, GA may inhibit ferroptosis induced by excessive exercise-related mitochondrial ROS production, thereby protecting against skeletal muscle injury [90]. Although this evidence was obtained from skeletal muscle rather than bone or joint tissues, it provides mechanistic support for the concept that excessive exercise-induced oxidative stress can promote ferroptosis when antioxidant defenses are overwhelmed.

It should be noted that the effects of exercise on ferroptosis are unlikely to be uniformly protective and may depend on exercise intensity, duration, and tissue context. Moderate aerobic exercise may enhance antioxidant defense, maintain iron homeostasis, and reduce lipid peroxidation, thereby decreasing susceptibility to ferroptosis. In contrast, prolonged or excessive exercise may disrupt iron homeostasis and generate persistent oxidative stress, which can promote lipid peroxidation and ferroptosis-related injury. Current evidence therefore supports a context-dependent, and potentially hormetic, relationship between exercise load and ferroptosis rather than a simple linear response. However, because existing studies differ substantially in species, tissues, disease models, and exercise protocols, a universal intensity or duration threshold separating protective from detrimental effects cannot yet be defined. Further studies are needed to clarify the dose-dependent and tissue-specific effects of different exercise modalities on ferroptosis in skeletal disorders.

### 5.3. Molecular Mechanisms of Ferroptosis in Aerobic Exercise-Mediated Improvement of Skeletal Disorders

**Figure 3:**
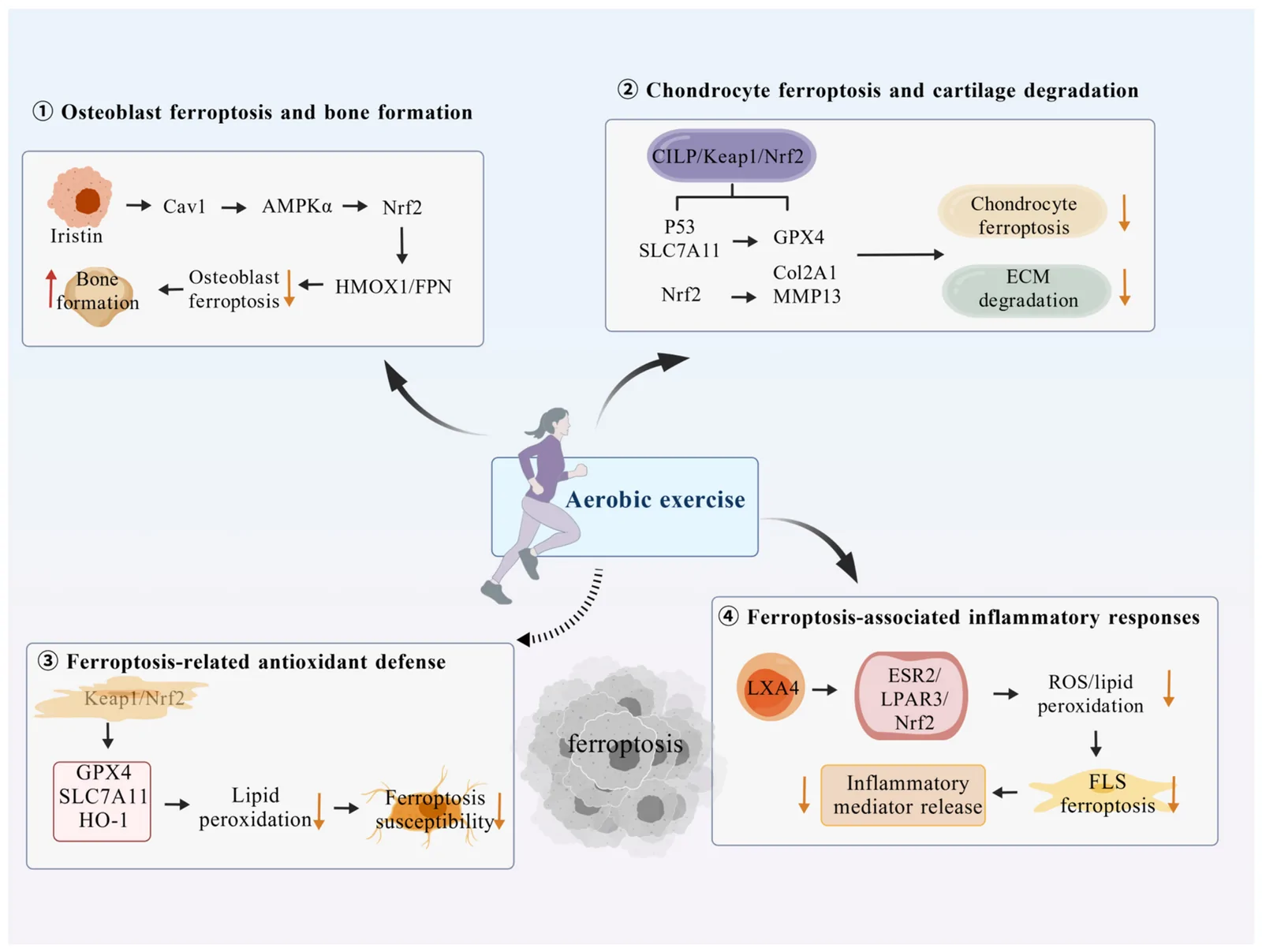
Ferroptosis-related mechanisms involved in the skeletal effects of aerobic exercise. In osteoblasts, exercise-induced irisin acts through Cav1–AMPKα–Nrf2 signaling, with subsequent regulation of heme oxygenase 1 (HMOX1) and FPN, thereby limiting osteoblast ferroptosis and supporting bone formation. In chondrocytes, moderate exercise regulates the p53/SLC7A11/GPX4 axis and the CILP/Keap1/Nrf2 pathway, which is associated with reduced chondrocyte ferroptosis and cartilage matrix degradation. Exercise may also strengthen ferroptosis-related antioxidant defense through Keap1/Nrf2-dependent regulation of GPX4, SLC7A11, and HO-1. In fibroblast-like synoviocytes (FLS), exercise-associated increases in LXA4 activate estrogen receptor 2 (ESR2)/lysophosphatidic acid receptor 3 (LPAR3)/Nrf2 signaling, reduce ROS and lipid peroxidation, and suppress ferroptosis and inflammatory mediator release. Solid curved arrows indicate pathways supported mainly by direct skeletal evidence, whereas the dashed curved arrow indicates a pathway supported in part by mechanistic evidence from non-skeletal tissues.

Current studies suggest that aerobic exercise may regulate ferroptosis through multiple molecular pathways. However, direct evidence from bone and joint tissues remains limited, and most available findings are derived from animal and cellular experiments. In addition, some proposed mechanisms are supported by studies conducted in non-skeletal tissues and therefore require further validation in skeletal cells and tissues (Figure 3; Table 2).

#### 5.3.1. Exercise-Mediated Regulation of Osteoblast Ferroptosis and Bone Formation

OBs are responsible for synthesizing and secreting bone matrix and play a central role in maintaining bone homeostasis [91]. Ferroptosis in OBs can impair their osteogenic capacity and contribute to bone loss. In experimental models of diabetic bone loss, high-glucose and high-fat conditions increase iron accumulation, reduce SLC7A11 and GPX4 expression, and impair osteoblast differentiation [92]. Although these findings were obtained without exercise intervention, they provide a mechanistic basis for understanding how regulation of osteoblast ferroptosis may influence bone formation.

Irisin is an exercise-induced myokine that can act on bone cells and promote osteogenesis [93]. More direct evidence comes from a recent study showing that 4 weeks of treadmill exercise increased skeletal muscle fibronectin type III domain-containing protein 5 (FNDC5)/irisin production and promoted its delivery to osteoblasts through myotube-derived exosomes [94]. After entering osteoblasts, irisin binds to caveolin-1 (Cav1), recruits AMPKα, and activates Nrf2 signaling. Nrf2 subsequently increases HMOX1 and ferroportin (FPN) transcription. HMOX1 contributes to osteoblast proliferation, whereas FPN promotes cellular iron efflux and reduces ferroptosis. Through this muscle–bone signaling pathway, aerobic exercise may therefore promote osteoblast proliferation and preserve bone formation by limiting ferroptosis [94].

At present, this represents relatively direct evidence linking aerobic exercise with ferroptosis regulation in osteoblasts. However, the evidence is still derived mainly from animal and cellular experiments, and whether the same mechanism contributes to the skeletal benefits of aerobic exercise in humans remains to be established.

#### 5.3.2. Exercise-Mediated Regulation of Chondrocyte Ferroptosis and Cartilage Matrix Degradation

The cartilage extracellular matrix (ECM), which is mainly maintained by chondrocytes, is essential for preserving joint structure and function, and progressive ECM degradation is a major pathological feature of OA [95]. IL-1β is an important inflammatory stimulus in OA and can induce chondrocyte dysfunction and cartilage matrix degradation [96]. Increasing evidence indicates that ferroptosis participates in this process. Glutathione peroxidase 7 (GPX7) overexpression reduces IL-1β-induced lipid peroxidation, Fe^2+^ accumulation, and ECM degradation in chondrocytes, while pharmacological inhibition of ferroptosis can also alleviate IL-1β-induced chondrocyte injury [97,98]. NF-κB signaling is also closely involved in inflammatory responses and pathological cartilage remodeling [99]. These studies provide a mechanistic basis for considering ferroptosis as one of the cellular processes involved in cartilage degeneration.

More importantly, direct exercise-related evidence has now been reported in OA models. Moderate-intensity treadmill exercise increases Col2A1 expression and reduces MMP13 expression in articular cartilage, while increasing SLC7A11 and GPX4 and reducing p53 expression. Complementary mechanical-stimulation experiments further showed that moderate mechanical stress activates Nrf2 signaling and modulates NF-κB p65 activity, thereby affecting the p53/SLC7A11/GPX4 axis and reducing chondrocyte ferroptosis [77]. These changes are accompanied by reduced ECM degradation and attenuation of OA progression.

A more recent study further identified cartilage intermediate layer protein (CILP) as an upstream regulator of this response. In an early OA rat model, moderate-intensity treadmill exercise increased CILP expression in articular cartilage. CILP interacts with Keap1, reduces Nrf2 ubiquitination, and promotes Nrf2 nuclear translocation, followed by increased expression of SLC7A11, HO-1, GPX4, and superoxide dismutase 1 (SOD1) [100]. These changes reduce lipid peroxidation and chondrocyte ferroptosis and are accompanied by attenuation of cartilage fibrosis and OA progression. Taken together, the available evidence suggests that moderate exercise may inhibit chondrocyte ferroptosis through both NF-κB/p53-related regulation and the CILP/Keap1/Nrf2 pathway [77,100]. However, these findings are currently based mainly on animal and cellular experiments, and their relevance to patients with OA requires further validation.

#### 5.3.3. Exercise-Regulated Ferroptosis and Antioxidant Defense

Oxidative stress and impaired antioxidant defense are closely associated with ferroptosis, and Nrf2-related signaling plays an important role in limiting lipid peroxidation and maintaining redox homeostasis [69]. As described above, studies in osteoblasts and chondrocytes have shown that aerobic exercise can influence Nrf2-associated antioxidant signaling together with ferroptosis-related molecules such as SLC7A11, GPX4, HO-1, and FPN [77,94,100]. These changes are consistent with the view that enhancement of antioxidant capacity is one of the mechanisms through which exercise may reduce ferroptosis susceptibility in skeletal tissues.

Similar responses have also been observed in non-skeletal tissues. In the prefrontal cortex of APP/PS1 mice, 8 weeks of aerobic exercise activated the System Xc^−^/GPX4 pathway and reduced ferroptosis associated with iron overload [74]. In hepatic experimental models, exercise intervention also reduced Fe^2+^ and MDA levels while increasing Nrf2, HO-1, SLC7A11, and GPX4 expression [101]. In aging rats, lifelong aerobic exercise regulated Keap1/Nrf2 signaling in skeletal muscle, increased GPX4 and SLC7A11 expression, and reduced ferroptosis-related oxidative damage [102]. Notably, the response differed between the quadriceps and soleus muscles, suggesting that exercise-induced regulation of ferroptosis may vary according to tissue or muscle fiber type.

These findings from brain, liver, and skeletal muscle provide additional support for the relationship between aerobic exercise, antioxidant defense, and ferroptosis. However, because these studies were not conducted in bone or joint tissues, they should be regarded as mechanistic evidence rather than direct proof that identical antioxidant responses occur in skeletal cells. At present, the most direct skeletal evidence remains concentrated in osteoblasts and chondrocytes.

#### 5.3.4. Exercise-Regulated Ferroptosis and Inflammatory Responses

Inflammation and ferroptosis are closely interconnected in skeletal disorders, particularly in OA. Lipoxin A4 (LXA4) is a pro-resolving lipid mediator involved in the regulation of inflammatory responses [103]. In experimental OA, treadmill exercise increases LXA4 levels and alleviates synovial inflammation and cartilage damage [104]. Further evidence indicates that LXA4 is also involved in the regulation of ferroptosis in fibroblast-like synoviocytes (FLS). In a rat model of knee OA, exercise-associated increases in LXA4 were accompanied by activation of the ESR2/LPAR3/Nrf2 pathway, reduced intracellular ROS and lipid peroxidation, and inhibition of ferroptosis in FLS [105]. These changes were associated with attenuation of synovial inflammation and OA progression. Thus, the LXA4/ESR2/LPAR3/Nrf2 pathway currently provides direct joint-specific evidence linking aerobic exercise, inflammatory regulation, and ferroptosis in OA.

STING may represent another link between inflammatory signaling and ferroptosis. Experimental studies have shown that STING/NF-κB signaling can influence bone homeostasis and osteogenic differentiation [106]. However, direct evidence linking exercise, STING-mediated ferroptosis, and skeletal disorders is still lacking. In a traumatic brain injury model, moderate-intensity treadmill exercise suppressed STING signaling, increased SLC7A11 and GPX4 expression, and reduced iron accumulation, lipid peroxidation, and ferroptosis [107]. This study supports a possible relationship between exercise-mediated STING regulation and ferroptosis, but because the evidence was obtained from neural tissue, the same mechanism cannot yet be assumed to operate in bone or joint cells. Overall, current evidence indicates that interactions between inflammatory signaling and ferroptosis may contribute to the skeletal effects of aerobic exercise. The LXA4/ESR2/LPAR3/Nrf2 pathway in OA is supported by direct joint-related evidence [104,105], whereas the involvement of STING is currently based mainly on bone-related background evidence and non-skeletal exercise models [106,107]. Further studies are needed to determine whether exercise regulates STING-dependent ferroptosis directly in skeletal tissues.

Beyond these pathway-specific uncertainties, several broader questions remain unresolved. It is not yet clear whether ferroptosis is a causal mediator of the skeletal benefits of aerobic exercise or an accompanying response to changes in oxidative stress and metabolism, nor whether mechanisms identified in specific tissues and disease models are shared across different skeletal disorders. Whether different exercise modalities, intensities, and durations produce distinct ferroptosis responses also remains to be determined.

The available exercise protocols also provide some indication of a dose-dependent relationship between exercise load and ferroptosis (Table 2). In direct skeletal models, ferroptosis-suppressive effects have been reported with treadmill exercise ranging from 10 to approximately 19.3 m/min, generally for 30–60 min/day and, where reported, 5 days/week for about 4 weeks [77,94,100,105]. Consistent protective responses have also been observed in non-skeletal models using moderate exercise intensities of approximately 50–80% VO_2_max [102,107]. In contrast, an excessive treadmill protocol of 25 m/min at a 15° incline for 60 min/day, 6 days/week for 8 weeks was associated with increased Fe^2+^ and MDA, reduced GPX4, and ferroptosis-related skeletal muscle injury [90]. Although these protocols are not directly comparable, the current evidence suggests that the shift toward a detrimental ferroptotic response may be associated with a greater cumulative exercise load—combining higher intensity, incline, frequency, and training duration—rather than with a single intensity or duration threshold.

**Table 2:** Evidence hierarchy and molecular mechanisms of exercise-mediated ferroptosis regulation in skeletal disorders.

Experimental Model	Target Tissue/Cell	Exercise Intervention	Ferroptosis-Related Markers/Pathways	Main Findings	Evidence Category	Year	Ref.
MIA-induced OA male SD rats and IL-1β-treated C28/I2 chondrocytes	Articular cartilage; chondrocytes	Moderate treadmill exercise: 18 m/min, 30 min/day, 5 days/week for 4 weeks	SLC7A11↑; GPX4↑; p53↓; Nrf2 signaling↑; NF-κB signaling↓	Treadmill exercise inhibits chondrocyte ferroptosis, reduces cartilage matrix degradation, and delays OA progression.	Direct skeletal evidence	2024	[77]
OVX female C57BL/6J mice and osteoblast/myotube models	Osteoblasts/bone tissue	Treadmill exercise, 10 m/min, 1 h/day for 4 weeks.	FNDC5/irisin↑; Cav1–AMPKα–Nrf2↑; HMOX1↑; FPN↑; GPX4↑; intracellular iron/lipid ROS↓	Aerobic exercise can alleviate bone loss in mice by promoting osteoblast proliferation and inhibiting ferroptosis.	Direct skeletal evidence	2024	[94]
ACLT-induced early OA rats and primary chondrocytes	Articular cartilage intermediate zone; chondrocytes	Moderate-intensity treadmill exercise at 19.2 m/min	CILP↑, Nrf2 nuclear translocation↑, SLC7A11↑, HO-1↑, GPX4↑, SOD1↑, MDA↓, GSH↑	Moderate-intensity treadmill exercise suppresses chondrocyte ferroptosis through activation of the CILP/Keap1/Nrf2 axis, contributing to reduced cartilage fibrosis and attenuation of early OA progression.	Direct skeletal evidence	2026	[100]
MIA-induced KOA rats and FLS–chondrocyte co-culture models	Synovium; fibroblast-like synoviocytes (FLSs)	Moderate treadmill exercise: 19.3 m/min, 5° incline, 60 min/day, 5 days/week for 4 weeks	LXA4↑; ESR2/LPAR3/Nrf2↑; GPX4↑; SOD1↑; ROS/lipid peroxidation↓	Aerobic exercise can promote the production of LXA4 and inhibit ferroptosis in FLS through the ESR2/LPAR3/Nrf2 axis, thereby alleviating the pathological progression of OA.	Direct skeletal evidence	2024	[105]
Excessive exercise-induced muscle injury in 8-week-old male mice and primary skeletal muscle cells	Skeletal muscle; skeletal muscle cells	Excessive treadmill exercise (25 m/min, 15° slope, 1 h/day, 6 days/week for 8 weeks)	Fe^2+^↑, MDA↑, COX2↑, GPX4↓, mitochondrial oxidative stress↑	Excessive exercise induces skeletal muscle injury associated with mitochondrial oxidative stress and ferroptosis activation, indicating a maladaptive response to exercise overload.	Indirect mechanistic evidence	2025	[90]
Naturally aging female SD rats	Quadriceps femoris; soleus muscle	Treadmill: 17 m/min (75–80% VO_2_max), 45 min/day, 5 days/week; LAT continued from 8 to 26 months of age	SES1↑, Nrf2↑, Keap1↓, GPX4 and SLC7A11↑, ACSL4↓	Lifelong aerobic exercise improves the antioxidant capacity of the quadriceps by activating the Keap1/Nrf2 pathway, thereby reducing susceptibility to ferroptosis.	Indirect mechanistic evidence	2023	[102]
CCI-induced TBI mice	Injured cerebral cortex; neurons	4 weeks of moderate-intensity treadmill exercise (30 min/day, 5 days/week; 10–15 m/min, 0°).	STING↓; SLC7A11/xCT↑; GPX4↑; ACSL4↓; Fe^2+^↓; lipid peroxidation↓; GSH↑	Aerobic exercise can inhibit STING-related signaling, increase GPX4 expression, reduce inflammatory factor release, and inhibit ferroptosis.	Indirect mechanistic evidence	2023	[107]

## 6. Conclusions

Aerobic exercise is an important non-pharmacological intervention for maintaining skeletal health and has beneficial effects on bone remodeling, cartilage homeostasis, oxidative stress, and inflammatory regulation. In this review, we summarized the major mechanisms of ferroptosis and its involvement in osteoporosis, osteoarthritis, rheumatoid arthritis, and osteonecrosis of the femoral head. We further discussed the emerging relationship between aerobic exercise and ferroptosis regulation, with particular attention to osteoblast function, cartilage matrix degradation, antioxidant defense, and inflammatory responses. Taken together, ferroptosis may contribute to some of the skeletal adaptations induced by aerobic exercise, although the strength of the supporting evidence varies across different skeletal disorders. 

However, the current evidence remains limited in several respects. Most mechanistic studies are derived from cellular and animal models, whereas direct evidence from human skeletal tissues and clinical exercise interventions is still scarce. Moreover, direct exercise–ferroptosis evidence in skeletal tissues is currently concentrated mainly in osteoblast-related bone loss and OA, whereas evidence for RA and ONFH remains largely indirect and does not yet establish ferroptosis as a mediator of exercise-induced effects in these diseases. Findings from skeletal muscle, cardiovascular, neural, and hepatic tissues provide useful mechanistic support but cannot be directly extrapolated to bone or joint tissues. In addition, the effects of exercise on ferroptosis appear to depend on exercise intensity, duration, tissue type, and physiological context, and the boundary between protective adaptation and excessive oxidative stress has yet to be clearly defined.

Future studies should therefore further clarify the tissue-specific mechanisms by which exercise regulates ferroptosis in different skeletal cell populations and determine whether these mechanisms are conserved in humans. Particular attention should be given to well-designed clinical studies that integrate exercise interventions with ferroptosis-related biomarkers in bone and joint tissues or circulation. Comparative studies of different exercise modalities, intensities, and durations are also needed to establish whether ferroptosis contributes to exercise-specific skeletal adaptations. Addressing these issues will provide new insights into the role of exercise in regulating ferroptosis and offer a more solid theoretical basis for developing exercise-based strategies to improve skeletal health.

## Data Availability

No new data were created or analyzed in this study. Data sharing is not applicable to this article.

## Abbreviations

ACSL4	Acyl-CoA synthetase long-chain family member 4
AGEs	Advanced glycation end products
AMPK	Adenosine monophosphate-activated protein kinase
AMPKα	Adenosine monophosphate-activated protein kinase alpha
AMPKα2	Adenosine monophosphate-activated protein kinase alpha 2
APP/PS1	APPswe/PSEN1dE9 mouse model
ATP	Adenosine triphosphate
BDNF	Brain-derived neurotrophic factor
BMSCs	Bone marrow mesenchymal stem cells
cGAS	Cyclic GMP-AMP synthase
Cav1	Caveolin-1
Col2A1	Collagen type II alpha 1 chain
DHODH	Dihydroorotate dehydrogenase
ECM	Extracellular matrix
ESR2	Estrogen receptor 2
Fer-1	Ferrostatin-1
FGF21	Fibroblast growth factor 21
FGFR1	Fibroblast growth factor receptor 1
FLS	Fibroblast-like synoviocytes
FPN	Ferroportin
FtMt	Mitochondrial ferritin
GLUT1	Glucose transporter 1
GPX4	Glutathione peroxidase 4
GSH	Glutathione
GSH-Px	Glutathione peroxidase
HFD	High-fat diet
HIF-1α	Hypoxia-inducible factor-1 alpha
HO-1	Heme oxygenase-1
IL-1β	Interleukin-1 beta
Keap1	Kelch-like ECH-associated protein 1
LPCAT3	Lysophosphatidylcholine acyltransferase 3
LXA4	Lipoxin A4
MDA	Malondialdehyde
MIA	Monosodium iodoacetate
MMP13	Matrix metalloproteinase-13
NCOA4	Nuclear receptor coactivator 4
NF-κB	Nuclear factor-kappa B
NQO1	NAD(P)H quinone oxidoreductase 1
Nrf2	Nuclear factor erythroid 2-related factor 2
OA	Osteoarthritis
OBs	Osteoblasts
OCs	Osteoclasts
OCN	Osteocalcin
ONFH	Osteonecrosis of the femoral head
OP	Osteoporosis
OPG	Osteoprotegerin
PINP	Procollagen type I *N*-terminal propeptide
PTGS2	Prostaglandin-endoperoxide synthase 2
PUFA-PLs	Polyunsaturated fatty acid-containing phospholipids
RA	Rheumatoid arthritis
RA-FLS	Rheumatoid arthritis fibroblast-like synoviocytes
RANKL	Receptor activator of nuclear factor-kappa B ligand
ROS	Reactive oxygen species
SIRT6	Sirtuin 6
SLC7A11	Solute carrier family 7 member 11
SOD	Superoxide dismutase
STING	Stimulator of interferon genes
TFRC	Transferrin receptor 1
TNF	Tumor necrosis factor
TNF-α	Tumor necrosis factor-alpha
TrkB	Tropomyosin receptor kinase B
